# Pangenome-wide identification and analysis of the heat shock transcription factor gene family in tea plant (*Camellia sinensis*)

**DOI:** 10.3389/fpls.2026.1949069

**Published:** 2026-09-03

**Authors:** Quanlong Liu, Shanyuanrui Lin, Zhuolin Li, Renjie Yin, Xiaohan Liu, Yan Gao, Linna Ma, Hechen Li, Yaman Yin, Peng Liu, Jiaqi Zhang

**Affiliations:** 1 School of Life Sciences, North China University of Science and Technology, Tangshan, China; 2 School of Life Science and Technology, Northwestern Polytechnical University, Xi’an, Shaanxi, China

**Keywords:** Camellia sinensis, gene family evolution, heat shock transcription factor, pangenome, PEG-induced stress

## Abstract

Heat shock transcription factors (*Hsf*) are key regulators of plant responses to environmental stresses, but their intraspecific diversity and evolutionary dynamics in the tea plant remain insufficiently understood. In this study, we performed a pangenome-wide identification and systematic analysis of the *Hsf* gene family across 22 *Camellia sinensis* genomes. A total of 536 *Hsf* genes were identified, with gene numbers ranging from 15 in HD to 33 in SCZ, indicating substantial accession-level copy number variation. Phylogenetic analysis classified these genes into three major subfamilies, including Hsf-A, Hsf-B, and Hsf-C, among which Hsf-A was the largest group. Orthogroup-based pangenome analysis assigned the 536 *Hsf* genes to 21 orthogroups, including 7 core orthogroups and 14 dispensable orthogroups, suggesting that dispensable genes represent an important component of the tea *Hsf* repertoire. Gene duplication analysis showed that dispersed duplication and WGD/segmental duplication were the major forces driving *Hsf* family expansion, whereas proximal and tandem duplications contributed relatively little. Copy number variation was widespread among *Hsf* orthogroups, especially in dispensable orthogroups. Selection pressure analysis revealed that most homologous *Hsf* gene pairs were under purifying selection, although selection intensity differed among subfamilies. Transcriptome analysis of SCZ showed tissue-preferential expression patterns and PEG-responsive expression divergence. Notably, Hsf-B genes exhibited the highest average expression under PEG stress and occupied a central position in the PCC-based co-expression network. Several genes, including CSS0020980.1 and CSS0019914.1, were identified as candidate PEG-responsive *Hsf* genes. These results provide a comprehensive pangenome-level framework for understanding *Hsf* gene evolution and stress-responsive regulation in tea plant.

## Introduction

As one of the world’s most significant beverage crops, the tea plant (*Camellia sinensis*) has been cultivated for millennia and today represents a cornerstone of the global agricultural economy. The tea plant is native to Southeast Asia, East Asia, and the Indian subcontinent but is now widely grown across tropical and subtropical regions worldwide. Beyond its traditional role in the beverage industry, *C. sinensis* also holds substantial value in the pharmaceutical and nutraceutical sectors, owing to the diverse bioactive compounds present in its leaves. However, the cultivation and productivity of this economically important cash crop are increasingly threatened by global climate change (Denton et al., 2014). Rising temperatures, prolonged heatwaves, irregular rainfall, and more frequent extreme weather events adversely affect tea plant growth, bud development, and secondary metabolism (Biol, 2009). Heat and water stress can harden tea leaves, alter the biosynthesis of key flavor compounds, and ultimately reduce both yield and market quality.

Heat shock transcription factors (*Hsf*) represent a pivotal class of transcriptional regulators that orchestrate plant responses to a broad spectrum of environmental stresses, most notably high temperature. These factors function as major regulators of plant heat stress responses by binding to heat shock elements (HSEs) in the promoter regions of target genes, thereby activating the expression of HSP genes and other stress-responsive genes, including genes encoding antioxidant enzymes such as APXs (Tong et al., 2025). Structurally, plant *Hsf* proteins are characterized by a highly conserved N-terminal DNA-binding domain (DBD) comprising three α-helices and a four-stranded antiparallel β-sheet, which is indispensable for sequence-specific DNA recognition (Matthews et al., 2024). This DBD is flanked by oligomerization domains, including hydrophobic heptad repeats (HR-A/B), which facilitate homo- and hetero-oligomer formation among *Hsf* family members (Fang et al., 2023). Based on the structural features of the flexible linker region connecting the DBD and the HR-A/B domain, plant *Hsf* proteins have been phylogenetically classified into three major subfamilies: A, B, and C (Scharf et al., 2012). Class A *Hsf* proteins possess C-terminal activation domains known as AHA motifs. These motifs confer autonomous transcriptional activation activity. In contrast, class B and class C *Hsf* proteins generally lack AHA motifs. Class B *Hsf* proteins often function as transcriptional repressors or regulatory modulators, whereas the functions of class C *Hsf* proteins remain less well understood (Czarnecka-Verner et al., 2000).

In recent years, with the improvement of sequencing technologies and the development of bioinformatics, an increasing number of *Hsf* gene families have been identified in model plants and economically important crops, such as rice (Ikeda et al., 2011), *Arabidopsis thaliana* (Scharf et al., 2012), soybean (Fragkostefanakis et al., 2019), tea plant (Xiang et al., 2013), cotton (Kolmos et al., 2014), and tomato (Jiang et al., 2023a). These findings indicate that *Hsf* proteins are central regulators of plant stress-responsive regulatory networks and provide an important foundation for further investigation of *Hsf*-mediated stress responses in tea plants. Although multiple tea plant genomes have been sequenced in recent years and pangenome analyses have provided valuable resources for dissecting genetic diversity, structural variation, and agronomic trait differentiation in *C. sinensis*, a pangenome-based investigation of the *Hsf* transcription factor family has not yet been reported (Camacho et al., 2009; Li et al., 2021; Jiang et al., 2023b). Previous genome-wide studies of *Hsf* genes in tea plant were mainly based on a single reference genome. Such reference-dependent analyses may fail to capture the full repertoire of *Hsf* members across diverse tea germplasms, especially genes affected by presence/absence variation, copy number variation, structural variation, and specific duplication events. This limitation restricts our understanding of the intraspecific diversity, evolutionary dynamics, and potential functional divergence of *CsHsf* genes. Therefore, a pangenome-wide analysis of the *Hsf* gene family is necessary to comprehensively characterize *CsHsf* diversity, clarify its evolutionary expansion and conservation patterns, and identify candidate genes with thermotolerance. Such information will provide a more complete genetic framework for understanding *Hsf*-mediated heat stress responses and for improving climate resilience in tea plant breeding.

## Results

### Identification and phylogenetic analysis of the *Hsf* gene family in 22 *Camellia sinensis*

**Figure 1:**
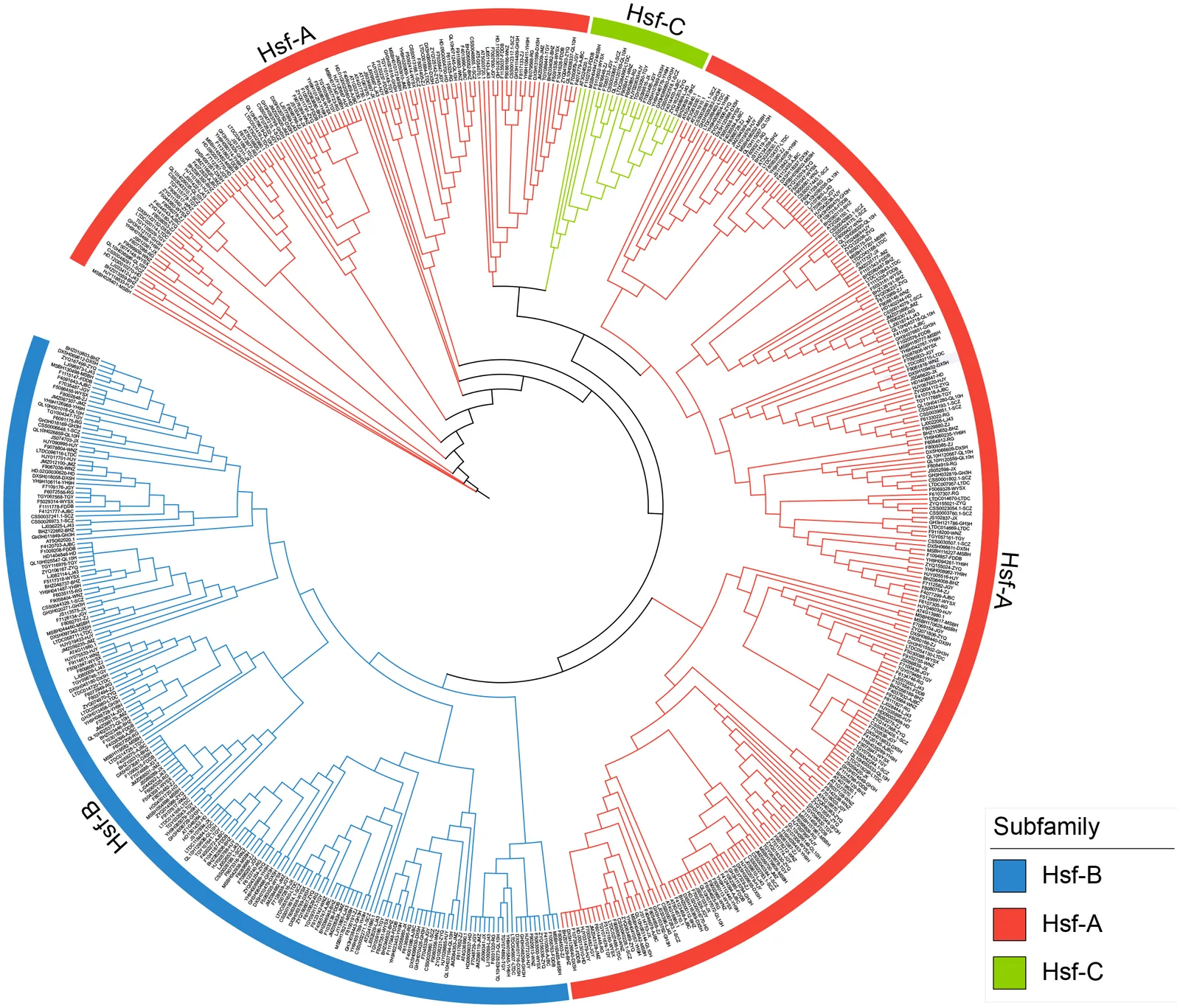
Phylogenetic tree constructed based on *Hsf* family genes from 22 *Camellia* accessions and *Arabidopsis thaliana*.

We downloaded 22 C*. sinensis* genomes, including AJBC, BHZ, DX5H, FDDB, G3GH, HD, HJY, JGY, JMZ, JX, LJ43, LTDC, MSBH, QL10H, RG, SCZ, TGY, WNZ, WYSX, YH9H, ZJ, and ZYQ. A total of 536 *Hsf* family genes were identified across these *C. sinensis* genomes, with the following counts: 25, 24, 25, 25, 25, 15, 22, 26, 18, 22, 22, 25, 22, 24, 30, 33, 22, 27, 26, 25, 26, and 27, respectively (**Supplementary Table 1**). Among them, SCZ harbored the largest *Hsf* gene repertoire (33 genes), while HD contained the fewest (15 genes), indicating substantial intraspecific variation in *Hsf* gene copy numbers. Subsequently, we performed multiple sequence alignment on 24 *Hsf* proteins from *Arabidopsis* and 536 *Hsf* proteins from *C. sinensis* plants, and constructed a phylogenetic tree. Based on the topology of the phylogenetic tree and the established subfamily classification system of *Arabidopsis*, the *Hsf* family was further divided into three subfamilies (Hsf-A, Hsf-B, and Hsf-C). Among these, Hsf-A was the largest subfamily, comprising 330 members (averaging 15 genes per accession), followed by Hsf-B with 184 members (averaging 8.3 genes per accession), while Hsf-C was the smallest subfamily, containing only 22 members (averaging 1 gene per accession) (**Figure 1**; **Supplementary Table 2**).

**Figure 2:**
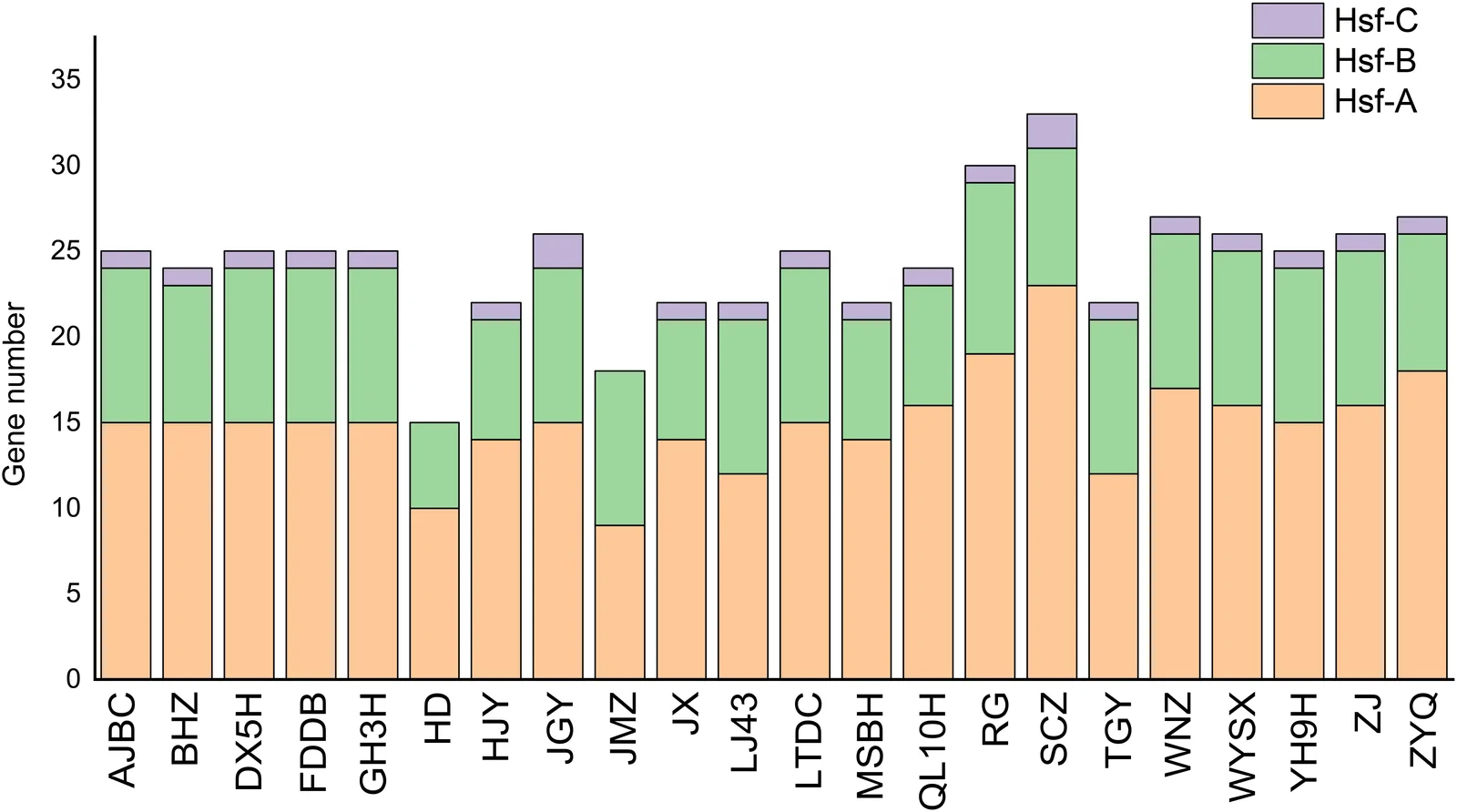
Number of genes in the three *Hsf* subfamilies across 22 accessions; accession abbreviations are shown on the x-axis.

Although the subfamily sizes are highly imbalanced across different *C. sinensis* accessions, most accessions retain members in every subfamily (except HD). In the Hsf-A subfamily, SCZ contained the highest number of genes (23), whereas JMZ contained the fewest (9). In the Hsf-B subfamily, RG had the largest number of genes (10), while HD had the smallest number (5). For the Hsf-C subfamily, the vast majority of the examined accessions contain only one copy of this gene (HD: 0, SCZ: 2) (**Figure 2**; **Supplementary Table 3**). Among them, the larger number of Hsf-A genes indicated a greater degree of copy number expansion in this subfamily. Hsf-C contained the fewest members. Most accessions retained one Hsf-C gene, whereas no Hsf-C gene was identified in HD and two copies were detected in SCZ.

### Physicochemical property analysis of the *Hsf* gene family in 22 *Camellia sinensis*

**Figure 3:**
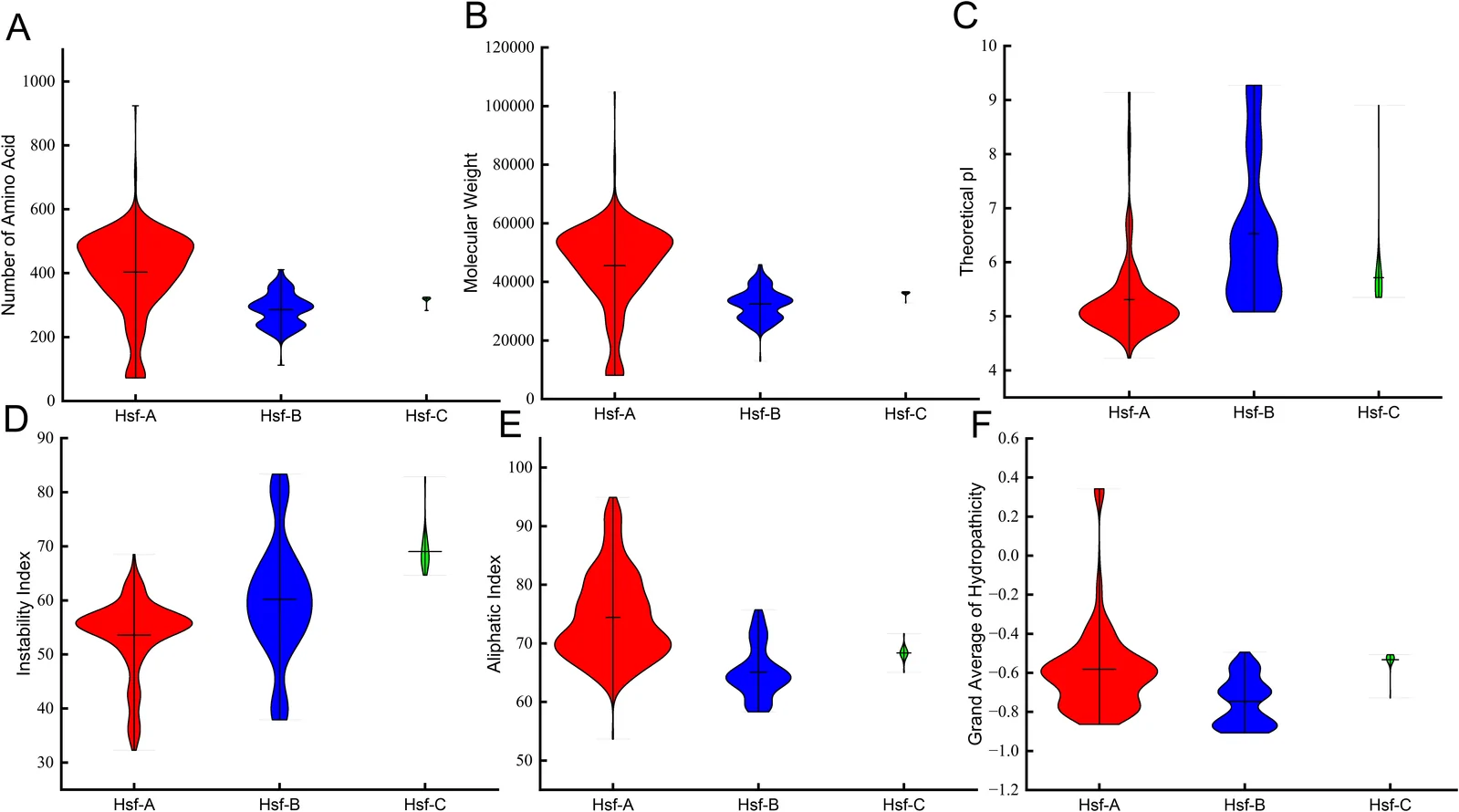
Physicochemical property analysis. **(A)** Number of amino acids in each *Hsf* subfamily. **(B)** Molecular weight in each *Hsf* subfamily. **(C)** Theoretical pI in each *Hsf* subfamily. **(D)** Instability index in each *Hsf* subfamily. **(E)** Aliphatic index in each *Hsf* subfamily. **(F)** Grand average of hydropathicity in each *Hsf* subfamily.

To clarify differences in *Hsf* gene structures among subfamilies in *C. sinensis*, we predicted and summarized the protein length, molecular weight (MW), theoretical isoelectric point (pI), instability index, aliphatic index, and grand average of hydropathicity (GRAVY) of 536 *CsHsf* proteins (**Figures 3A–F**; **Supplementary Table 4**). Overall, the amino acid lengths of *CsHsf* proteins ranged from 72 to 924 aa, their MWs ranged from 8.11 to 104.78 kDa, and their theoretical pI values ranged from 4.23 to 9.27, indicating that most *CsHsf* proteins were acidic. The aliphatic index ranged from 53.65 to 94.63. Based on the instability index, the majority of *CsHsf* proteins (94.21%) were predicted to be unstable, whereas only 5.79% were classified as stable (**Figures 3A–F**; **Supplementary Table 4**). In addition, most *CsHsf* proteins showed negative GRAVY values (97.67%), while only a small proportion showed positive values, suggesting that most *CsHsf* proteins were hydrophilic (**Figures 3A–F**; **Supplementary Table 4**).

Different *Hsf* subfamilies may exhibit distinct physicochemical characteristics. Therefore, we further summarized and compared the physicochemical properties of *CsHsf* proteins among the Hsf-A, Hsf-B, and Hsf-C subfamilies. The results revealed pronounced differences in several physicochemical parameters among these subfamilies (**Figures 3A–F**; **Supplementary Table 5**). The mean protein length was shortest in Hsf-B (approximately 285.6 aa), intermediate in Hsf-C (approximately 319.8 aa), and markedly longer in Hsf-A (approximately 403.7 aa) (**Figure 3A**). In terms of protein size, Hsf-A showed the widest MW range and the highest mean MW (8.11–104.78 kDa, mean ≈ 45.47 kDa), indicating substantial variation in sequence length within this subfamily. In contrast, Hsf-C displayed a highly concentrated MW distribution (32.81–36.63 kDa; mean approximately 36.17 kDa), consistent with its relatively conserved structural features (**Figure 3B**). Regarding theoretical pI, Hsf-A had the broadest pI range (4.23–9.14), whereas Hsf-B and Hsf-C showed relatively similar pI distributions, ranging from 5.08 to 9.27 and from 5.35 to 8.90, respectively (**Figure 3C**). In terms of the instability index, both the mean and median values were highest for Hsf-C (mean ≈ 69.04, median = 70.73) and lowest for Hsf-A (mean ≈ 53.59, median ≈ 54.90), indicating that Hsf-C exhibits the poorest stability. For the aliphatic index, Hsf-A exhibited the widest range, with values ranging from 53.65 to 94.63. Hsf-B ranged from 58.73 to 75.73, while Hsf-C ranged from 65.02 to 71.66 and had the lowest mean value (**Figure 3E**). For GRAVY, all positive values were observed in the Hsf-A subfamily, with values ranging from -0.864 to 0.342 and a mean of approximately -0.581. Among the three subfamilies, Hsf-B was the most hydrophilic, with GRAVY values ranging from -0.908 to -0.494 and a mean of approximately -0.745, whereas Hsf-C was relatively less hydrophilic, with GRAVY values ranging from -0.728 to -0.507 and a mean of approximately -0.532 (**Figure 3F**).

### Core and dispensable *Hsf* gene analysis in 22 *Camellia sinensis* genomes

**Figure 4:**
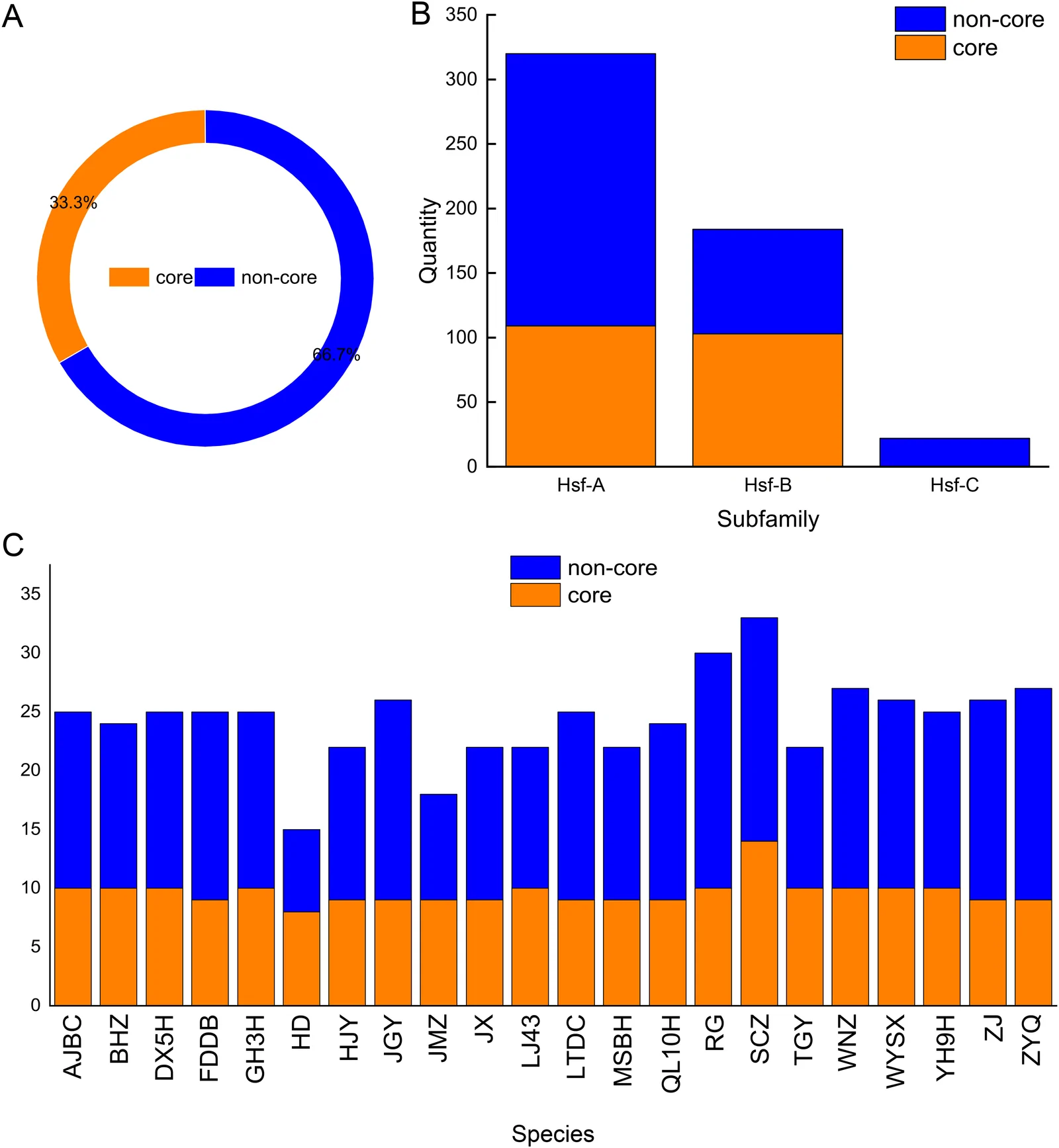
Core and dispensable *Hsf* gene analysis. **(A)** Number and proportion of core and dispensable orthogroups in the *Hsf* family. **(B)** Numbers of core and non-core genes in each subfamily of the *Hsf* family. **(C)** Numbers of core and non-core genes in 22 *Camellia* accessions.

To investigate *Hsf* family genes in *C. sinensis* from a homology perspective, OrthoFinder was used to cluster the 536 *Hsf* genes into orthogroups. In total, 536 genes were assigned to 21 orthogroups (OGGs) (**Supplementary Table 7**), including 7 core orthogroups containing 212 genes (39.5%) and 14 dispensable orthogroups containing 324 genes (60.5%) (**Figure 4A**; **Supplementary Table 7**). Statistical analysis showed that the mean amino acid length was 374 aa for core genes and 350 aa for non-core genes (**Supplementary Table 6**). For molecular weight, the mean value of core genes was 42.09 kDa. Although non-core genes had a lower mean molecular weight (39.64 kDa), their maximum value (104.78 kDa) was markedly higher than that of core genes (78.11 kDa). The mean theoretical pI was 5.48 for core genes and 5.91 for non-core genes (**Supplementary Table 6**). Regarding the instability index, the mean value was higher in core genes than in non-core genes (59.56 vs 54.52), indicating that core genes were relatively more unstable. For the aliphatic index, the mean value was 69.36 in core genes and 72.03 in non-core genes. In terms of GRAVY, all core genes had negative values, with a mean of -0.68, whereas a small number of non-core genes had positive values, with an overall mean of -0.60 (**Supplementary Table 6**).

The differences in core and non-core gene numbers among subfamilies were also pronounced (**Figure 4B**; **Supplementary Table 8**). In the Hsf-A subfamily, there were 109 core genes (35.2%) and 201 non-core genes (64.8%). The Hsf-B subfamily had a relatively high proportion of core genes (103, 55.9%), indicating that it is comparatively conserved and undertakes some essential functions, while the non-core genes numbered 81 (44.1%). As for the Hsf-C subfamily, all genes were non-core (22, 100%), suggesting that it experiences weaker selective constraints (**Figure 4B**; **Supplementary Table 8**). We next performed a chi-square test of independence on the 3 × 2 contingency table of subfamily versus core/non-core status (**Supplementary Table 9**). The results indicated a significant association between core and non-core genes, rejecting independence (χ^2^ ≈ 41.03, df = 2, p ≈ 12.3 × 10^-9^, N_total = 536) (**Supplementary Table 9**). However, Cramer’s V was 0.277, suggesting only a moderately weak association, implying that subfamily membership is only one of many factors influencing core/non-core status (**Supplementary Table 9**). To address concerns about expected frequencies below 5 (particularly for Hsf-C), we also performed a permutation test with 20, 000 permutations, which likewise yielded a significant association (p ≈ 5.0 × 10^-5^) (**Supplementary Table 9**). Subfamily-specific Fisher’s exact tests (with BH-FDR correction) revealed that the distribution of core/non-core genes differed significantly across all three subfamilies (FDR < 0.05) (**Supplementary Table 10**). Hsf-A showed a significant bias toward non-core genes (OR = 0.493); Hsf-B exhibited a significant enrichment of core genes (OR = 2.835, with the highest core proportion), indicating it is relatively most conserved; and Hsf-C consisted entirely of non-core genes (OR = 0). These results demonstrate that the distribution of core and non-core genes is non-random and biased, with Hsf-C in particular being composed exclusively of specific genes (**Supplementary Table 10**).

HD contained 15 core and non-core *Hsf* genes in total, which was the lowest number among the 22 accessions (**Figure 4C**; **Supplementary Table 11**). These results showed that HD had the smallest *Hsf* gene repertoire among the examined accessions. Except for SCZ, which contains 14 core genes, all other accessions have 9 or 10 core genes. RG has the highest number of non-core genes, suggesting that RG may possess more specific genes or has experienced relatively more gene expansion events (**Figure 4C**; **Supplementary Table 11**).

### Duplication types and CNV analysis of the *Hsf* gene family

**Figure 5:**
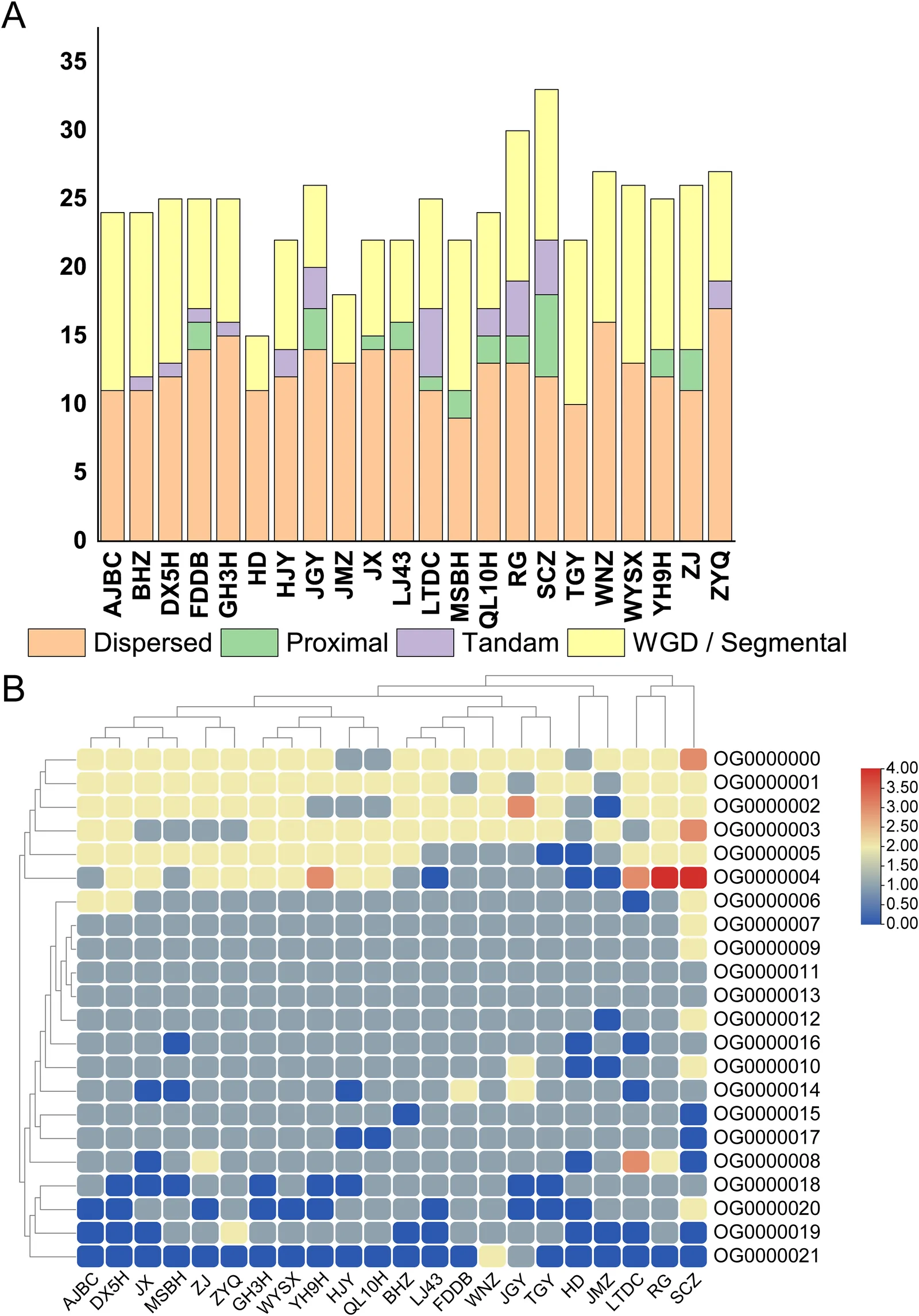
Duplication types and CNV analysis. **(A)** The number of genes for each duplication type across 22 *Camellia* accessions. **(B)** Heatmap of copy number variation (CNV).

To investigate the expansion mechanisms of the *C. sinensis Hsf* gene family, we summarized the duplication types of *Hsf* genes across 22 tea accessions (**Figure 5A**; **Supplementary Table 12**). Duplication types were assigned to all 536 *Hsf* genes. Overall, *Hsf* gene family expansion was mainly driven by dispersed duplications (278, 51.87%) and WGD/segmental duplications (205, 38.25%), whereas proximal (27, 5.04%) and tandem duplications (26, 4.85%) contributed only slightly. Notably, no singleton genes were detected (**Figure 5A**; **Supplementary Table 12**). Clear differences in duplication patterns were observed among the 22 tea accessions. Proximal duplications accounted for the highest proportion in SCZ (6/33, 18.18%), while tandem duplications were relatively frequent in RG and SCZ, accounting for 13.33% (4/30) and 12.12% (4/33), respectively. AJBC and LTDC showed the lowest proportions of dispersed duplications, both with 44.00% (11/25) (**Figure 5A**, **Supplementary Table 12**).

Duplication patterns also differed markedly among *Hsf* subfamilies (**Supplementary Table 13**). In the Hsf-A subfamily, dispersed duplications accounted for the highest proportion (175, 53.03%), followed by WGD/segmental duplications (114, 34.55%), whereas proximal and tandem duplications represented only minor proportions (19, 5.76% and 22, 6.67%, respectively) (**Supplementary Table 13**). In the Hsf-B subfamily, WGD/segmental duplications were the most abundant, reaching 89 copies and accounting for 48.37%, followed closely by dispersed duplications (85, 46.19%). Proximal and tandem duplications accounted for only small proportions, with 8 proximal duplicates (4.35%) and 2 tandem duplicates (1.09%) (**Supplementary Table 13**). In the Hsf-C subfamily, dispersed duplications were predominant (18, 81.82%), suggesting that this subfamily may have undergone more extensive dispersed duplication-associated divergence. Tandem and WGD/segmental duplications each accounted for only two copies (9.09%), and no proximal duplications were detected in this subfamily (**Supplementary Table 13**).

From the perspective of core and non-core orthogroups, WGD/segmental duplication was the predominant duplication type in core orthogroups, accounting for 64.15% of genes (136/212), followed by dispersed duplication (70/212, 33.02%) (**Supplementary Table 14**). Only a small number of proximal and tandem duplications were observed in core orthogroups, with 5 and 1 genes, respectively, accounting for 2.36% and 0.47%. In contrast, dispersed duplications accounted for the highest proportion in non-core orthogroups (208/324, 64.20%), followed by WGD/segmental duplications (69/324, 21.29%) (**Supplementary Table 14**). Proximal and tandem duplications were more frequent in non-core orthogroups than in core orthogroups, with 22 and 25 genes, respectively, accounting for 6.79% and 7.72%. The predominance of dispersed duplications in non-core orthogroups suggests that these genes may be more prone to lineage-specific expansion and functional divergence than core genes (**Supplementary Table 14**). In contrast, the dominance of WGD/segmental duplications in core orthogroups indicates that these genes may have been retained after ancient polyploidization or large-scale segmental duplication events.

Copy number variation (CNV), an important type of structural variation in plants with pronounced functional consequences, can influence phenotypic diversity and promote evolutionary divergence. In multi-accession comparisons, expansion and contraction of gene families are often reflected by CNV. Based on OrthoFinder analysis of the *Hsf* family across 22 C*. sinensis* accessions, most orthogroups (OGGs) showed variable copy numbers among accessions (20/22, 90.90%), indicating widespread CNV within the *Hsf* gene family (**Figure 5B**; **Supplementary Table 7**). Among the seven core orthogroups, marked differences in copy number expansion were observed. Specifically, OG0000000 and OG0000001 each maintained two copies in most accessions, accounting for 81.82% (18/22) and 86.36% (19/22) of the accessions, respectively (**Figure 5B**; **Supplementary Table 7**). In contrast, OG0000011 and OG0000013 uniformly retained a single copy across all accessions, suggesting that these orthogroups may be relatively conserved. Compared with core orthogroups, the 15 non-core orthogroups showed stronger presence/absence variation, copy-number fluctuation, and accession-specific expansion (**Figure 5B**; **Supplementary Table 7**). For example, OG0000021 was detected only in WNZ and JGY, with two and one copies, respectively, whereas OG0000004 reached a maximum of four copies in SCZ and RG and also contained three copies in JMZ and YH9H (**Figure 5B**; **Supplementary Table 7**). Together, these results indicate pronounced expansion and contraction divergence of the *Hsf* gene family among *C. sinensis* accessions.

### Selection pressure analysis of the *Hsf* gene family

**Figure 6:**
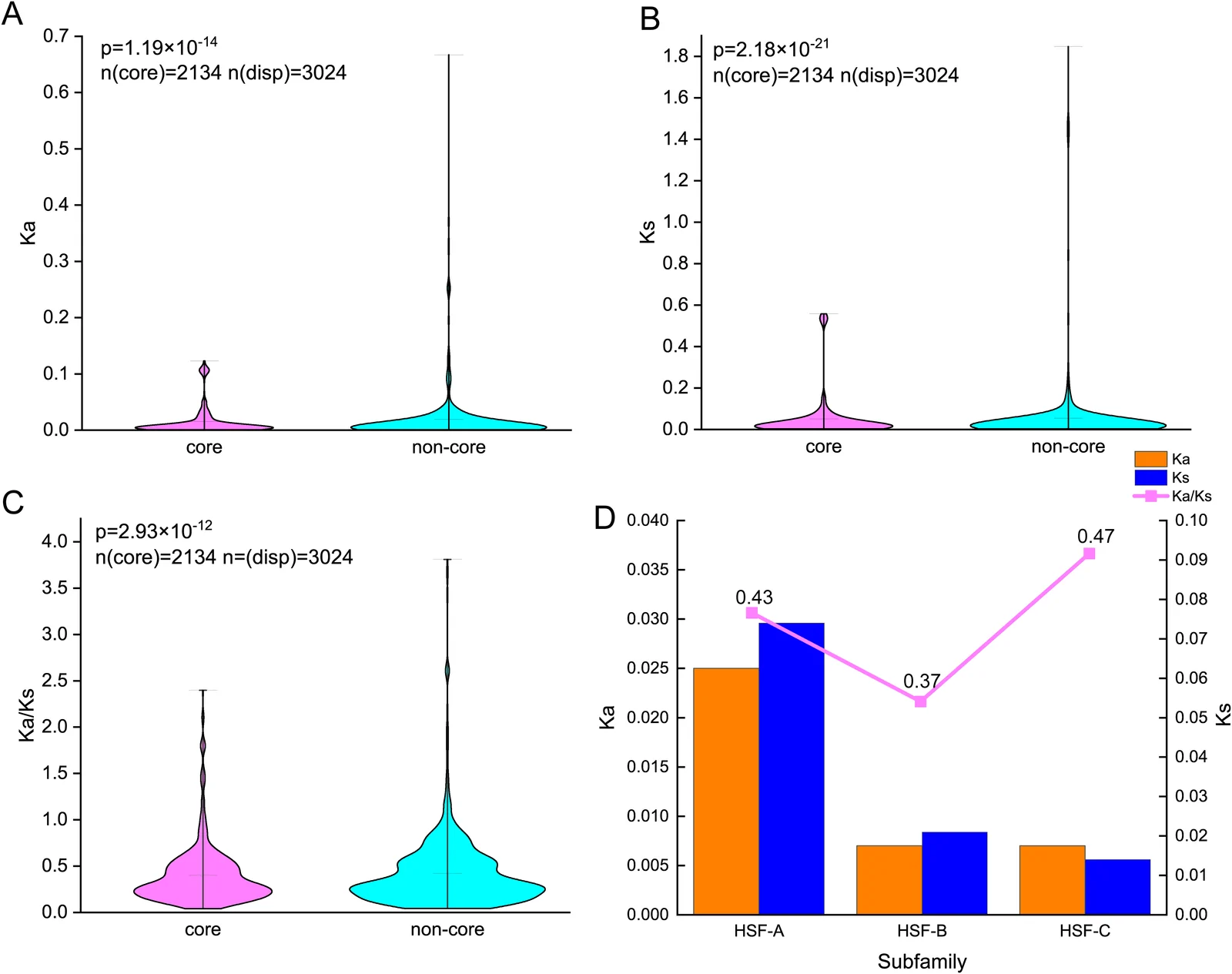
Selection pressure analysis. **(A)** Ka values of core and non-core genes. **(B)** Ks values of core and non-core genes. **(C)** Ka/Ks values of core and non-core genes. **(D)** Ka, Ks, and Ka/Ks values for each subfamily of the *Hsf* family.

To further investigate selective pressure on the *Hsf* gene family across 22 C*. sinensis* accessions, we calculated Ka/Ks ratios for 9, 306 gene pairs derived from 22 OGGs (**Figures 6A–C**; **Supplementary Table 19**). Among the 5, 158 valid orthologous gene pairs retained for analysis, the vast majority (4, 920 pairs, 95.39%) exhibited Ka/Ks ratios below 1, whereas only 238 pairs (4.61%) showed Ka/Ks ratios above 1 (**Figures 6A–C**; **Supplementary Table 19**). The median values of Ka, Ks, and Ka/Ks were 0.005, 0.019, and 0.288, respectively, indicating that most *Hsf* genes were under purifying selection. The corresponding mean values were 0.019, 0.054, and 0.423, respectively (**Figures 6A–C**; **Supplementary Table 19**). Significant differences were detected between core and non-core orthogroups (t-test, p < 0.01) (**Figures 6A–C**; **Supplementary Table 20**). Compared with non-core gene pairs, core gene pairs showed higher Ka and Ka/Ks values, suggesting that they may have experienced relatively weaker selective constraints. Although both groups were mainly subject to purifying selection, the Ka/Ks ratio of the core group was closer to 1, indicating a tendency toward relaxed purifying selection rather than strict neutral evolution (**Figures 6A–C**; **Supplementary Table 20**).

We next calculated the average Ka, Ks, and Ka/Ks values for each *Hsf* subfamily across the 22 C*. sinensis* accessions (**Figure 6D**; **Supplementary Table 19**). The mean Ka/Ks ratios of all three subfamilies were below 1, ranging from 0.372 to 0.477, confirming that the *Hsf* gene family was primarily shaped by purifying selection at the sequence level, although the intensity of selection varied among subfamilies. Among the three subfamilies, Hsf-C showed the highest mean Ka/Ks ratio (Ka ≈ 0.008, Ks ≈ 0.015, Ka/Ks ≈ 0.477) (**Figure 6D**; **Supplementary Table 19**), indicating the weakest selective constraint, a greater potential to accumulate nonsynonymous substitutions, and a higher likelihood of functional divergence. Hsf-A ranked second, with mean Ka, Ks, and Ka/Ks values of approximately 0.025, 0.075, and 0.433, respectively (**Figure 6D**; **Supplementary Table 19**). The lowest mean Ka/Ks value was observed in Hsf-B (Ka ≈ 0.007, Ks ≈ 0.022, Ka/Ks ≈ 0.372), suggesting that this subfamily experienced the strongest purifying selection and may have retained a relatively high degree of sequence conservation.

### Transcriptome analysis of the *Hsf* gene family

**Figure 7:**
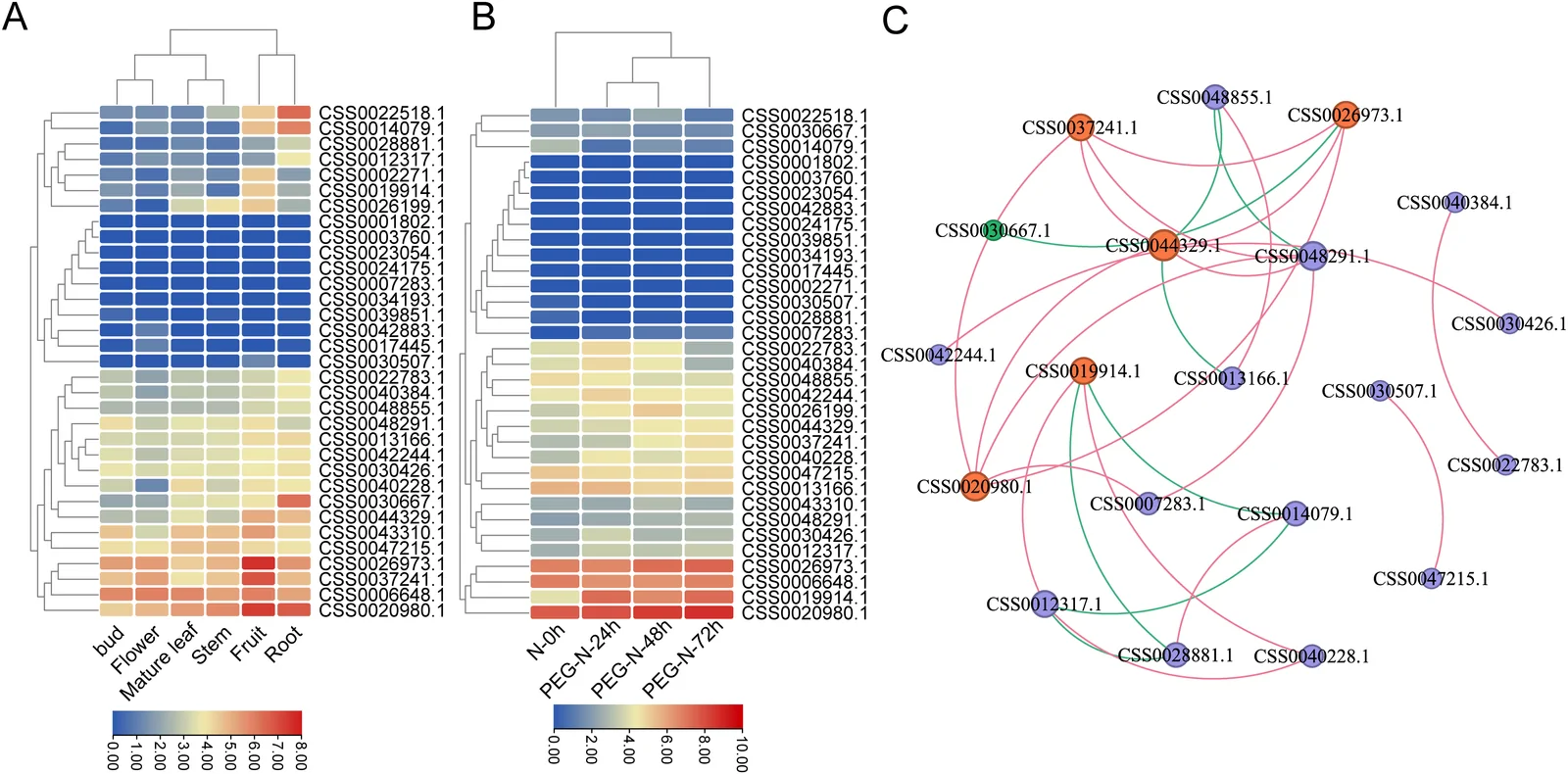
Transcriptome analysis. **(A)** Heatmap of expression levels of *Hsf* genes in various tissues of SCZ. **(B)** Heatmap of expression levels of *Hsf* genes in SCZ under PEG-induced stress. **(C)** PCC (Pearson correlation coefficient) analysis of potential co-expression relationships among *Hsf* genes in SCZ under PEG-induced stress conditions.

To investigate the expression divergence of SCZ *Hsf* genes, the expression values were transformed using log2(x+1) prior to analysis. In tissue-specific expression profiles, SCZ *Hsf* genes showed clear tissue-preferential expression patterns, with the highest average expression observed in fruit (3.15), followed by root (2.97), mature leaf (2.27), stem (2.23), bud (2.05), and flower (1.91) (**Figure 7A**; **Supplementary Table 20**). Comparison among subfamilies revealed that Hsf-B genes exhibited the highest overall tissue expression level, with an average value of 4.02 across all tissues, followed by Hsf-C (3.26) and Hsf-A (1.80). In particular, Hsf-B genes showed strong expression in fruit (5.80) and root (4.19), whereas Hsf-C genes were most highly expressed in root (6.35) and fruit (4.20), suggesting potential tissue-specific functional divergence among *Hsf* subfamilies (**Figure 7A**; **Supplementary Table 20**). At the gene level, CSS0020980.1, an Hsf-B member, showed the highest average expression across tissues (5.74) and was highly expressed in fruit (7.36), root (6.69), and stem (5.75) (**Figure 7A**; **Supplementary Table 20**). Another Hsf-B gene, CSS0026973.1, also displayed prominent fruit-biased expression, with the highest expression value in fruit (7.63), indicating that several Hsf-B members may be preferentially active in fruit development or fruit-related physiological processes. In contrast, Hsf-A genes generally maintained relatively low expression across tissues, with slightly higher expression in root (2.25) and fruit (2.13) (**Figure 7A**; **Supplementary Table 20**). Under PEG-induced stress conditions, the average expression of SCZ *Hsf* genes increased from 2.54 at 0 h to 2.75 at 24 h and reached the highest level at 48 h (2.77), followed by a slight decrease at 72 h (2.69), indicating a moderate stress-responsive expression pattern (**Figure 7B**; **Supplementary Table 19**). Subfamily-level analysis further showed that Hsf-B genes were the most strongly expressed under stress, increasing continuously from 4.43 at 0 h to 4.98, 5.31, and 5.45 at 24, 48, and 72 h, respectively (**Figure 7B**; **Supplementary Table 19**). Consistently, CSS0020980.1 maintained the highest expression level under PEG stress and increased from 7.66 at 0 h to 8.73 at 72 h, suggesting a stable and strong response to PEG treatment. In addition, CSS0019914.1, another Hsf-B member, showed the most obvious induction pattern, increasing from 4.08 at 0 h to 7.29 at 24 h and 7.31 at 72 h. By contrast, Hsf-A genes showed only a slight transient increase at 24 h, while Hsf-C genes displayed relatively weak and unstable responses, with decreased expression at 72 h (**Figure 7B**; **Supplementary Table 19**).

To further explore potential co-expression relationships among SCZ *Hsf* genes under PEG-induced stress, pairwise Pearson correlation coefficient (PCC) analysis was performed based on their PEG-responsive expression profiles (**Figure 7C**; **Supplementary Table 20**). Gene pairs with strong correlations were retained using an absolute PCC threshold of |PCC| ≥ 0.95. In total, the resulting co-expression network contained 20 nodes and 27 edges, including 19 positive co-expression edges and 8 negative co-expression edges (**Figure 7C**; **Supplementary Table 20**). The predominance of positive edges suggested that most strongly correlated SCZ *Hsf* gene pairs exhibited similar expression trends under PEG stress, whereas negative edges indicated opposite expression patterns between specific gene pairs. Among all retained genes, CSS0044329.1 showed the highest connectivity, with six correlated edges, including four positive and two negative associations, suggesting that it may act as a central co-expression hub in the PEG-responsive *Hsf* network. CSS0020980.1 and CSS0048291.1 also showed high connectivity, each with five edges (**Figure 7C**; **Supplementary Table 20**). In contrast, several genes, including CSS0022783.1, CSS0030426.1, CSS0030507.1, CSS0030667.1, CSS0040384.1, CSS0042244.1, and CSS0047215.1, had only one retained edge, indicating relatively limited co-expression connectivity under the applied threshold. At the subfamily level, Hsf-A members accounted for the largest number of retained nodes, with 14 genes present in the network, followed by Hsf-B with five genes and Hsf-C with one gene (**Figure 7C**; **Supplementary Table 20**). However, Hsf-B genes showed the highest average connectivity, with an average degree of 4.60, substantially higher than that of Hsf-A genes (2.14) and Hsf-C genes (1.00). Notably, all six Hsf-B–Hsf-B edges were positive, indicating highly coordinated expression among Hsf-B members under PEG stress. In addition, Hsf-A–Hsf-A and Hsf-A–Hsf-B interactions each contributed ten edges, suggesting that both intra-subfamily and inter-subfamily co-expression relationships were involved in the PEG stress response (**Figure 7C**; **Supplementary Table 20**). These results indicate that Hsf-B members may occupy a more central position in the SCZ *Hsf* co-expression network under PEG-induced stress, while Hsf-A members contribute broadly to network complexity through both positive and negative co-expression relationships.

## Discussion

In this study, we performed a pangenome-wide identification and systematic analysis of the *Hsf* gene family across 22 *Camellia sinensis* genomes. A total of 536 *Hsf* genes were identified and classified into three major subfamilies, Hsf-A, Hsf-B, and Hsf-C, with Hsf-A representing the largest group (**Figures 1**, **2**; **Supplementary Table 1**). Considerable copy-number variation was observed among tea accessions, with SCZ harboring the largest *Hsf* repertoire and HD containing the fewest members (**Figure 4A**; **Supplementary Table 12**). Core/dispensable gene analysis revealed that non-core genes accounted for a larger proportion of the *Hsf* family, whereas Hsf-B showed the highest proportion of core genes, suggesting stronger evolutionary conservation (**Figure 4B**; **Supplementary Table 8**). Duplication and CNV analyses further indicated that dispersed and WGD/segmental duplications were the major forces driving *Hsf* expansion (**Figure 5B**; **Supplementary Table 7**). Most *Hsf* genes were under purifying selection, although selection intensity differed among subfamilies. Transcriptome and PCC analyses suggested that Hsf-B members were highly expressed and centrally connected under PEG-induced stress, highlighting their potential importance in stress-responsive regulation (**Figure 7C**; **Supplementary Table 20**).

### Pangenome diversity of the *Hsf* gene family in tea plant

Plant *Hsf* genes have been widely studied in model plants and crops, but most previous analyses were based on a single reference genome. Tea plant *Hsf* studies have also been largely limited to individual reference genomes. A previous study identified 25 *CsHsf* genes from a single tea reference genome and demonstrated the role of *CsHsfA2* in heat tolerance (Xiang et al., 2013). In contrast, our analysis identified 536 *Hsf* genes across 22 tea accessions, with 15 to 33 members in each accession. This variation could not be fully represented by a single reference genome. SCZ contained the largest *Hsf* repertoire, whereas HD contained the fewest members. The smaller number of *Hsf* genes in HD may reflect accession-specific gene loss or differences in its evolutionary history. However, variation in genome assembly completeness and gene annotation quality may also affect the number of genes identified in each accession (Denton et al., 2014). Further sequence-level validation is needed before the smaller Hsf repertoire in HD can be interpreted as a true biological contraction.

Previous tea pangenome studies showed that the 22 accessions represent broad genetic diversity and provide valuable resources for gene discovery and genomics-assisted breeding (Jiang et al., 2023b). Structural variation and presence or absence variation can also influence gene expression and agronomic traits in tea plant (Jones et al., 2014). Consistent with these findings, our analysis identified a high proportion of dispensable *Hsf* genes and extensive copy number variation among *Hsf* orthogroups. These results indicate that variation in gene content is an important component of *Hsf* diversity among tea accessions. Core *Hsf* genes were retained across all 22 accessions and may represent the broadly conserved component of the tea *Hsf* family. In contrast, dispensable genes were absent from one or more accessions and contributed more strongly to variation in gene content. Their high proportion suggests that a substantial part of the tea *Hsf* repertoire is variable within the accessions. This variation may provide genetic material for differences in stress responses among tea accessions, although direct associations with stress-related traits remain to be established. Overall, these findings show that analysis of a single reference genome may underestimate the diversity of the *CsHsf* family, particularly for genes affected by variation in presence and copy number.

### Evolutionary trajectories of tea *Hsf* genes

Gene duplication was an important contributor to the evolution of the tea *Hsf* family. A previous pangenome study of the tea TCP family also identified WGD/segmental and dispersed duplication as major sources of gene family expansion (Li et al., 2021). In our study, WGD/segmental duplication was enriched in core *Hsf* orthogroups, whereas dispersed duplication predominated in non-core orthogroups. These contrasting patterns suggest that core and non-core *Hsf* genes followed different evolutionary trajectories. The enrichment of WGD/segmental duplicates in core orthogroups suggests that many conserved *Hsf* genes were retained after large-scale genome or segmental duplication events. The retention of duplicated transcription factors may help preserve gene dosage and regulatory balance within transcriptional networks (Biol, 2009). In contrast, dispersed duplication may have contributed more strongly to recent gene gain and copy number variation among tea accessions. This pattern may explain why dispersed duplicates were more common in the variable component of the *Hsf* family. Most *Hsf* gene pairs had Ka/Ks values below 1, indicating that purifying selection was the dominant evolutionary force acting on the tea *Hsf* family. However, core gene pairs showed higher Ka and Ka/Ks values than non-core gene pairs. This result appears unexpected because core genes are present in all accessions and are often assumed to be more strongly conserved (Tong et al., 2025). Core status, however, describes the distribution of a gene across genomes and does not necessarily indicate stronger sequence conservation. One possible explanation is that core orthogroups contained a higher proportion of WGD and segmental duplicates (Matthews et al., 2024). These duplicated genes may remain present across all accessions because of dosage requirements, while individual copies gradually accumulate sequence or regulatory divergence (Fang et al., 2023). Differences in duplication age may also contribute to the observed pattern. Therefore, the higher Ka/Ks values of core *Hsf* genes may reflect relatively relaxed purifying selection in some retained duplicates rather than positive selection.

### Subfamily-specific conservation and functional divergence

Subfamily-level comparisons revealed distinct evolutionary patterns among Hsf-A, Hsf-B, and Hsf-C. Hsf-A was the largest subfamily and contained the highest proportion of non-core genes. Dispersed duplication was also the predominant duplication type in this group. These patterns indicate that Hsf-A experienced greater copy number expansion and accession-level variation than the other subfamilies. The expansion of Hsf-A may have increased the opportunity for functional diversification after gene duplication. However, subfunctionalization and neofunctionalization cannot be inferred from gene number alone and require further functional evidence. Hsf-B showed a different evolutionary pattern. It contained the highest proportion of core genes and had the lowest mean Ka/Ks ratio among the three subfamilies. WGD and segmental duplication also contributed strongly to this group. These results suggest that many Hsf-B genes have been retained across tea accessions and remain relatively conserved at the sequence level. Class B *Hsf* proteins generally lack the AHA activation motif and often function as transcriptional repressors or regulatory modulators (Czarnecka-Verner et al., 2000; Scharf et al., 2012). However, a repressive function does not imply a minor role in stress responses. A highly expressed repressor may occupy a central position in a regulatory network by limiting excessive transcription and controlling the timing or intensity of stress responses (Ikeda et al., 2011; Fragkostefanakis et al., 2019). Therefore, the high expression and connectivity of Hsf-B genes under PEG treatment may reflect a regulatory buffering function rather than direct transcriptional activation (Xiang et al., 2013; Kolmos et al., 2014). Hsf-B proteins may regulate the activity or expression of Hsf-A genes, modulate downstream HSP expression, and contribute to the restoration of transcriptional balance during prolonged osmotic stress. This hypothesis may explain the prominent position of Hsf-B genes in the PEG response, but it requires experimental validation. Hsf-C contained only 22 members, and one copy was retained in most tea accessions. No Hsf-C gene was identified in HD, whereas two copies were present in SCZ. Therefore, the classification of Hsf-C genes as non-core mainly reflects their absence from at least one accession and does not indicate that they are accession-specific. Hsf-C also showed the highest proportion of dispersed duplicates and the highest mean Ka/Ks ratio among the three subfamilies. These patterns may indicate greater sequence divergence or weaker selective constraint in this group. However, this interpretation should remain cautious because Hsf-C contained far fewer genes than Hsf-A and Hsf-B. Genome completeness and gene annotation quality may also have affected the apparent absence of Hsf-C in HD.

### Stress-responsive expression and candidate *Hsf* genes

Expression and co-expression analyses revealed distinct responses of SCZ *Hsf* genes to PEG treatment and identified several potential candidate genes. Previous studies have shown that plant *Hsf* proteins regulate responses to heat, drought, and osmotic stress (Fragkostefanakis et al., 2019; Xia et al., 2019). Overexpression of *GmHsf-34* improved drought and heat tolerance in *Arabidopsis*, indicating that some *Hsf* genes function in both temperature and dehydration responses (Fragkostefanakis et al., 2019). In tea plant, *CsHsfA2* has been associated with heat tolerance (Xiang et al., 2013). Other *CsHsfA* genes may also connect heat responses with flavonoid regulation through the *CsHsfA* and CsJAZ6 module (Camacho et al., 2009; Jiang et al., 2023a; Jiang et al., 2023b). Phytohormone signaling may also contribute to stress responses mediated by *Hsf* proteins. ABA, jasmonic acid, salicylic acid, ethylene, auxin, and brassinosteroids participate in plant stress responses and may influence *CsHsf* expression through interactions among hormone signaling pathways. Further experiments are needed to examine this possibility in tea plant (Li et al., 2021). In our study, Hsf-B genes showed the highest average expression under PEG treatment. They also had the highest average connectivity in the co-expression network. Among these genes, CSS0020980.1 and CSS0019914.1 showed notable but distinct expression patterns. CSS0020980.1 maintained a high expression level throughout the treatment, whereas CSS0019914.1 showed a stronger increase after PEG exposure. These different patterns suggest that the two genes may contribute to different stages or aspects of the osmotic stress response. Further experiments are required to determine their specific functions.

## Materials and methods

### Data sources

The genome sequences, protein sequences, coding sequences, and annotation files of 22 tea plant (*C. sinensis*) accessions were downloaded from the Tea-Pangenome database (https://www.tea-pangenome.cn/) (Chen S. et al., 2023). These accessions included AJBC, BHZ, DX5H, FDDB, G3GH, HD, HJY, JGY, JMZ, JX, LJ43, LTDC, MSBH, QL10H, RG, SCZ, TGY, WNZ, WYSX, YH9H, ZJ, and ZYQ. To identify members of the *Hsf* gene family, the hidden Markov model (HMM) profile corresponding to the heat shock transcription factor domain was obtained from the InterPro database (Jones et al., 2014). The transcriptome expression data of the SCZ accession, including tissue-specific expression profiles and PEG-induced stress expression profiles, were obtained from the Tea Plant Information Archive (TPIA; http://tpia.teaplants.cn/) database (Xia et al., 2019; Gao et al., 2024).

### *Hsf* family identification and physicochemical property analysis

To comprehensively identify *Hsf* gene family members in 22 tea plant genomes, a combined BLASTP- and hidden Markov model (HMM)-based strategy was performed. First, the whole-genome protein sequences of each tea accession were extracted from the corresponding genome annotation files, and only the longest protein isoform was retained for each gene when alternative transcripts were present. Known *Hsf* protein sequences from *Arabidopsis thaliana* were collected and used as queries to search against the tea plant protein databases using BLASTP with an E-value cutoff of 1e-5 (Camacho et al., 2009). In parallel, the HMM profile of the *Hsf-type* DNA-binding domain (PF00447) was downloaded from the InterPro database and used to scan the tea plant proteomes with HMMER 3.0 using an E-value threshold of 1e-5. Candidate *Hsf* proteins obtained from the BLASTP and HMM searches were merged, and redundant sequences were removed. All candidate proteins were then submitted to InterProScan for domain confirmation (Jones et al., 2014). The physicochemical properties of the confirmed *CsHsf* proteins, including amino acid length, molecular weight, theoretical isoelectric point, instability index, aliphatic index, and grand average of hydropathicity, were predicted using the Protein Parameter Calc module in TBtools-II (Chen C. et al., 2023).

### Phylogenetic analysis of *Hsf* genes

To clarify the evolutionary relationships and subfamily classification of the *Hsf* gene family in tea plant, the 536 *Hsf* protein sequences identified from 22 C*. sinensis* genomes were combined with previously reported *A. thaliana Hsf* protein sequences for phylogenetic analysis. Multiple sequence alignment of all *Hsf* protein sequences was performed using MAFFT v7.475 (Katoh et al., 2019). The aligned sequences were then used to infer a maximum-likelihood phylogenetic tree with IQ-TREE v2.4.0, and the optimal amino acid substitution model was automatically selected during tree construction. Branch reliability was estimated with 1, 000 bootstrap replicates (Minh et al., 2020). Based on the phylogenetic topology and the clustering relationships with well-characterized *A. thaliana Hsf* members, the *C. sinensis Hsf* proteins were assigned to three major subfamilies, namely Hsf-A, Hsf-B, and Hsf-C, according to the established plant *Hsf* classification system (Zhang et al., 2020). The final phylogenetic tree was visualized and annotated using iTOL (Letunic and Bork, 2024).

### Pangenome analysis of the *Hsf* gene family

To investigate the conservation and variation of the *Hsf* gene family across tea plant accessions, orthogroup-based pangenome analysis was performed using OrthoFinder v2.5.4 (Emms and Kelly, 2019). Following the pangenome-based gene family classification strategy described in a previous bHLH pangenome study, OGGs present in all 22 tea genomes were defined as core orthogroups, whereas OGGs absent from at least one genome were classified as dispensable orthogroups (Tong et al., 2025).

### Gene duplication-type analysis of *Hsf* genes

To characterize the duplication patterns contributing to the expansion of the *Hsf* gene family in tea plant, genome-wide duplication-type classification was performed for each of the 22 C*. sinensis* genomes. First, all protein sequences from each genome were subjected to all-versus-all sequence similarity searches using DIAMOND with an E-value cutoff of 1e-5 (Buchfink et al., 2015). The resulting similarity files, together with the corresponding genome annotation files converted into MCScanX-compatible format, were used as input for MCScanX to detect collinear blocks and classify gene duplication types (Wang et al., 2012). Genome-wide duplication categories were assigned using the duplicate_gene_classifier program implemented in MCScanX. According to the genomic distribution and collinearity relationships of duplicated genes, all genes were classified into five duplication types: singleton, dispersed, proximal, tandem, and WGD/segmental duplicates.

### Selection pressure analysis of *Hsf* genes

To evaluate the selection pressure acting on the *Hsf* gene family across 22 C*. sinensis* genomes, the coding sequences and corresponding protein sequences of the identified *Hsf* genes were used for Ka/Ks analysis. Homologous *Hsf* gene pairs were extracted from each orthogroup based on the OrthoFinder clustering results. For each homologous gene pair, the nonsynonymous substitution rate (Ka), synonymous substitution rate (Ks), and Ka/Ks ratio were calculated using the Simple Ka/Ks Calculator module in TBtools-II (Chen C. et al., 2023).

### PCC-based co-expression network analysis

To investigate the co-expression relationships among SCZ *Hsf* genes under PEG-induced stress, Pearson correlation coefficient (PCC) analysis was performed based on their expression profiles using a custom Python script (Zainal-Abidin et al., 2022). Prior to correlation analysis, the expression values of SCZ *Hsf* genes under PEG treatment were transformed using log2(x+1). Pairwise PCC values were calculated for all *Hsf* gene pairs, and only strongly correlated gene pairs with an absolute correlation coefficient of |PCC| ≥ 0.95 were retained for network construction. The resulting co-expression network was imported into Gephi for visualization and network topology analysis (Bastian et al., 2009).

## Data Availability

The original contributions presented in the study are included in the article/**Supplementary Material**. Further inquiries can be directed to the corresponding authors.
